# Silencing of P2Y_2_ receptor delays Ap_4_A*-*corneal re-epithelialization process

**Published:** 2009-06-11

**Authors:** Almudena Crooke, Aránzazu Mediero, Ana Guzmán-Aránguez, Jesús Pintor

**Affiliations:** Departamento de Bioquímica, E.U. Óptica, Universidad Complutense de Madrid, Madrid, Spain

## Abstract

**Purpose:**

There are no selective antagonists for the metabotropic nucleotide P2Y_2_ receptor subtype. This implies that it is not possible to demonstrate the importance of such a receptor in the relevant process of corneal wound healing. Therefore, we have cloned and designed a small interference RNA (siRNA) against the rabbit *P2Y_2_* receptor (*P2Y_2_-R*) mRNA, which clearly demonstrates the importance of this receptor in the process of wound healing triggered by nucleotides and dinucleotides both in vitro and in vivo.

**Methods:**

Rabbit *P2Y_2_-R* cDNA was cloned using a combination of degenerate reverse transcription polymerase chain reaction (RT-PCR) and rapid amplification of cDNA ends (RACE). To test the efficacy of synthesized siRNAs targeting *P2Y_2_-R*, immunocytochemistry, immunohistochemistry, and quantitative RT-PCR (qRT–PCR) assays were performed. Migration assays were performed both in vitro and in vivo by wounding the epithelium with a pipette tip and n-heptanol, respectively. These wounds were performed 72 h after siRNA transfection either in the presence or the absence of the P2Y_2_ agonist, 100 μM Ap_4_A (diadenosine tetraphosphate).

**Results:**

The cloned receptor presents 93% homology compared to the human gene. Two siRNAs were designed and synthesized against a rabbit *P2Y_2_-R* sequence. After transfection (in vitro assays) or topical instillation (in vivo assays), we demonstrated *P2Y_2_-R* siRNA efficient transfection/delivery and its efficient gene silencing. Clear reduction of P2Y_2_-R expression was observed at both the mRNA and protein levels in corneas treated with siRNA. In vitro and in vivo migration analysis showed that the silencing process has concomitantly reduced the ability of corneal cells to close the wounds in the presence of the Ap_4_A. In addition, both synthesized siRNAs exert a delay effect on the Ap_4_A-induced migration rate in vitro. These results suggest the absence of non-specific (off-target) effects by our siRNA.

**Conclusions:**

The application of *P2Y_2_-R* siRNA has demonstrated the role of this receptor in the accelerating effect of diadenosine tetraphosphate (Ap_4_A) on the corneal wound healing process.

## Introduction

Epithelial cell repair is a biochemical process which relies on the activation of intracellular pathways triggered by receptors stimulated by extracellular messengers. In the cornea, this physiologic process (corneal epithelial renewal) is essential to form a regular refractive surface and to form a barrier that contributes to corneal deturgescence and the prevention of pathogen invasion [1,2]. The renewal of the corneal epithelium is sustained by the migration, proliferation, and differentiation of stem cells present in the limbus.

Many substances present in tears or the aqueous humor or released from corneal nerves stimulate the wound healing process after ocular surface injuries [3,4]. Since the corneal surface is frequently damaged by refractive surgery, chemical trauma, inflammation, or infection, mechanisms by which epithelial receptors and its ligands elicit control of corneal epithelial renewal/wound healing have been investigated.

Among the naturally occurring substances recently found to modify the rate of healing, nucleotides and dinucleotides present in tears have been shown to be very active [4,5]. Nucleotides influence the corneal epithelial cells healing rate by either accelerating or decelerating the healing process. The way the wound healing process takes place depends on the metabotropic P2 receptors that are activated. It has been tentatively suggested that the activation of P2Y_2_ receptors accelerates the rate of corneal epithelial cell migration while the stimulation of a P2Y_6_ does the opposite [6]. The positive role of the P2Y_2_ receptor (P2Y_2_-R) is a matter of interest in understanding how this receptor triggers the intracellular machinery that produces an increase in the rate of healing. However, due to the lack of selective antagonists [7-9], it is not possible to prove the involvement of P2Y_2_-R in the corneal wound healing process.

RNA interference (RNAi) technology has proven to be an efficient tool to suppress the expression of targeted genes in vitro and, more recently, in vivo. In vivo assays have allowed us to evaluate the therapeutic potential of synthetic small interfering RNAs (siRNAs). For a review of in vivo use of siRNA, see [10]. Ocular delivery of siRNAs has also been performed for both basic biology research and to develop new treatments for ocular diseases [11,12].

The lack of selective antagonists to block the action of P2Y_2_-R as previously commented led us to investigate the role of P2Y_2_-R in corneal wound healing by designing a siRNA capable to eliminate the expression of this protein in corneal cells. Therefore, in this experimental work, we describe the cloning of rabbit *P2Y_2_-R* and the design of a siRNA against *P2Y_2_-R* mRNA, which clearly demonstrates the importance of this receptor in the process of wound healing triggered by nucleotides and dinucleotides both in vitro and in vivo.

## Methods

### Animals

Male, adult New Zealand White rabbits were used in these studies. All the animals were kept in individual cages with free access to food and water under controlled cycles (12 h light:12 h dark), and the experimental procedures were performed in accordance with the ARVO Statement for the Use of Animals in Ophthalmic and Vision Research and the European Communities Council Directive (89/609/EEC).

### Corneal epithelial cell culture

The rabbit corneal cell line, SIRC (Statens Seruminstitut Rabbit Cornea), was obtained from ATCC (LGC Promochem SL., Barcelona, Spain). SIRC cells were maintained in minimum essential medium (MEM) with Earle’s salts, L-glutamine, and non-essential amino acids (Invitrogen, Paisley, UK) supplemented with 10% activated FBS (Invitrogen), and incubated at 37 °C in 5% CO_2_ and 95% humidity until confluence.

### Cloning and sequencing

The rabbit *P2Y_2_* receptor cDNA was cloned using a combination of degenerate reverse transcription polymerase chain reaction (RT-PCR) and rapid amplification of cDNA ends (RACE). Total RNA was extracted from SIRC cells using the RNeasy Mini Kit (Qiagen, Barcelona, Spain). For first-strand cDNA synthesis, 5 µg of total RNA was retrotranscribed using the SuperScript III Reverse Transcriptase and oligo(dT; Invitrogen. The initial PCR was performed with a pair of degenerate primers based on two highly conserved regions of *P2Y_2_-R* sequences (forward, 5′-TGC AAG CTG GTG CGY TTC CTY TTC TA-3′ and reverse, 5′-AGY CTC TGC CCW GCC AGG AAG TAG AG-3′). The PCR amplification was performed in a 50 µl volume with 2 µl of cDNA, 1X PCR buffer, 2 mM MgCl_2_, 200 µM each dNTPs, 0.6 µM of each primer, and 0.025 U/µl of AmpliTaq Gold® DNA polymerase (Applied Biosystems, Foster City, CA). The thermal cycling conditions for PCR were 95 °C for 5 min; 5 cycles of 95 °C for 45 s and 72 °C for 2 min each; 5 cycles of 95 °C for 45 s, 70 °C for 45 s, and 72 °C for 2 min each; and 25 cycles of 95 °C for 45 s, 64 °C for 45 s, and 72 °C for 2 min each.

The PCR products of the expected size were extracted from 1.5% low-melt agarose gels with the QIAquick Gel Extraction Kit (Qiagen), cloned with TOPO TA Cloning Kit (Invitrogen), and then sequenced. After sequencing, a new pair of primers was synthesized (forward, 5′-TCA ACG AGG ACT TCA AGT AYG T-3′ and reverse, 5′-CTG ATA CAA GTG AGG AAG AGG AT-3′) and used to obtain contiguous sequence information. PCR amplification was performed in a 50 µl volume with 2 µl of cDNA, 1X PCR buffer, 2 mM MgCl_2_, 200 µM each dNTP, 0.4 µM of each primer, and 0.025U/µl of AmpliTaq Gold® DNA polymerase (Applied Biosystems). The thermal cycling conditions for PCR were 95 °C for 5 min; 10 cycles of 95 °C for 45 s, 60 °C for 45 s (−1 °C/cycle), and 72 °C for 1 min each; 30 cycles of 95 °C for 45 s, 50 °C for 45 s, and 72 °C for 1 min each; and 1 cycle of 72 °C for 7 min each.

To obtain the 5′-end of the coding sequence of rabbit *P2Y_2_-R* cDNA, we used a FirstChoice RLM-RACE Kit (Applied Biosystems). In brief, 10 µg of total RNA was dephosphorylated, decapped, and ligated to the 5′-RACE adaptor. Ligated RNA was reverse transcribed with random decamers and amplified by nested PCR. The first round of PCR was performed with a rabbit *P2Y_2_-R* specific reverse primer (5′-AGT GGT CGC GGG CGT AGT AGT AG-3′), and a forward adaptor primer provided in the kit. One µl of the obtained PCR product was used as a template for a second round of PCR. The second round of PCR was performed with a new rabbit *P2Y_2_-R* specific reverse primer (5′-ACA CGG CCA GGT GGA ACA TGT A-3′) and an inner forward adaptor primer provided in the kit. The PCR products of the expected size were purified from agarose gels, cloned, and sequenced. DNA sequencing was performed by the Unidad de Genómica (Parque Científico de Madrid-Universidad Complutense, Madrid, Spain). The nucleotide sequences were compared by searching the GenBank databases with the BLAST program. Alignment of amino acid sequences were performed with the Clustal W2 program [13] using default parameters.

**Figure 1:**
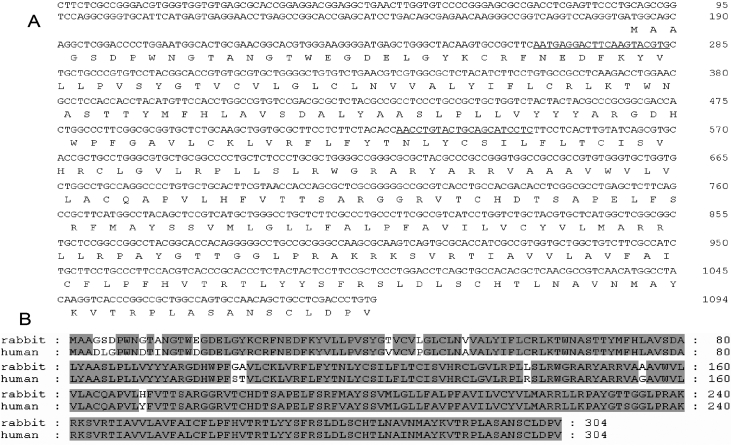
Rabbit *P2Y_2_-R* cDNA. **A**: Nucleotide sequence and deduced amino acid sequence of cloned rabbit P2Y_2_-R are shown. siRNA-target sequences are indicated with solid lines. **B**: Alignment of the rabbit deduced amino acid sequence with the human P2Y_2_-R sequence is displayed. Identical amino acids are shaded. Protein database searches revealed that the deduced sequence had the highest amino acid identity with human P2Y_2_-R (93%). These sequences are available under GenBank accession number EU886321 (rabbit) and NP_788086.1 (human).

To design *P2Y_2_-R* specific siRNA duplexes, the rabbit *P2Y_2_-R* coding sequence (GenBank EU886321) was submitted to the Ambion siRNA target Finder website for siRNA prediction. Two sequences of nine (maximum GC content 60%) suggested candidates were selected. Nucleotide sequences of the siRNA target sites were as follows: *P2Y_2_-R* siRNA #1, 5′-AAT GAG GAC TTC AAG TAC GTG-3′ (see Figure 1A) and *P2Y_2_-R* siRNA #2, 5′-AAC CTG TAC TGC AGC ATC CTC-3′ (Figure 1A). Both siRNAs were obtained from Applied Biosystems in annealed and lyophilized forms and were suspended in RNase-DNase-free water or in 0.9% NaCl before in vitro or in vivo use. In addition, a Cy3-labeled non-silencing siRNA, used to determine optimal conditions for siRNA transfection, was also purchased from Applied Biosystems. Transfection complexes were prepared in an Opti-MEM serum free medium (Invitrogen) by mixing 3 µl of siPORT NeoFX transfection reagent (Applied Biosystems) and 300 nM of *P2Y_2_-R* siRNA or just in RNase-DNase-free water (transfection reagent alone was the control). SIRC cells (50,000 cells per well) were plated in a 24 well format after the addition of transfection complexes. Cells were then incubated for 72 h and analyzed by migration assay. To evaluate transfection efficiency, SIRC cells were incubated for 24 h with 30 nM Cy3-labeled non-silencing siRNA, washed briefly in PBS, and then fixed in 4% paraformaldehyde for 15 min. Nuclei localization was analyzed using PicoGreen staining (1:200). PicoGreen (Invitrogen) is a fluorescent DNA-specific dye that has been recently used to analyze DNA distribution in permeabilized cells [14,15]. Finally, cells were visualized by a confocal microscope (Axiovert 200M; Carl Zeiss Meditec GmbH, Jena, Germany), equipped with a Pascal confocal module (LSM 5; Carl Zeiss Meditec GmbH). Cell viability was assessed by means of the trypan blue test (Sigma-Aldrich, St. Louis, MO ).

### Immunocytochemistry

SIRC cells were transfected for 72 h with the *P2Y_2_-R* siRNA or  the transfection reagent alone. The cells were then washed with 10X PBS and fixed for 15 min at room temperature with 4% paraformaldehyde in 0.15 M PBS. Cells were washed again with 10X PBS and permeabilized with blocking solution (1X PBS, 3% BSA, Triton X-100, 5% FBS) for 1 h at room temperature to block the non-specific binding sites. Cells were then washed with 1X PBS and 3% BSA and incubated with primary goat polyclonal anti-P2Y_2_-R (1:50; Santa Cruz Biotechnology, Santa Cruz, CA) or with 1X PBS and 3% BSA for negative controls at room temperature for 1 h. Cells were washed twice in 1X PBS and 3% BSA  and incubated with the secondary antibody donkey anti-goat IgG-FITC (1:200; Santa Cruz Biotechnology) for 1 h at room temperature. Finally, three washes in 1X PBS were performed, and coverslips were applied to the slides with Vectashield mounting medium (Vector Laboratories Inc., Burlingame, CA). The cells were observed by confocal microscope (Axiovert 200M; Carl Zeiss Meditec GmbH) equipped with a Pascal confocal module (LSM 5; Carl Zeiss Meditec GmbH). All images were managed with the accompanying Pascal software.

### Cell wounding and migration assays

After transfection for 72 h with *P2Y_2_-R* siRNA or transfection reagent alone, cell monolayers were wounded and treated with 100 µM of diadenosine tetraphosphate (Ap_4_A; Sigma-Aldrich) as previously described [6]. Briefly, SIRC confluent monolayers were wounded by scraping the cell monolayer with a pipette tip. The initial wound size and shape were comparable (60,000–80,000 μm^2^) to take into account the variations in wound closure due to size, so healings shared the same mechanistic features as previously indicated by other authors [16]. Wound area measurements (for each treatment) were collected from four different wells and averaged as one measurement (mean±SEM.). Cells were challenged with the dinucleotide dose for 2 min in Locke medium to avoid interference from components of media such as DMEM [17]. After this incubation, cells were washed and fresh MEM was added. A control experiment (cells transfected with the transfection reagent alone) was performed by challenging wounded monolayers with Locke medium (repeated 4 independent times). The dinucleotide dose was added to the wound when wound was first made (0 h) and 6 h after, as previously described for rabbits [4]. Images were captured every 2 h for the first 10 h and at 24 h after the beginning of the experiment. Wounds were measured with LSM 5 Pascal software (Zeiss Axiovert 200M microscope). Between different image collections, cells were kept in the CO_2_ incubator at 37 °C in 5% CO_2_ and 95% humidity.

### In vivo delivery of *P2Y_2_-R* siRNA and wounding procedure

The siRNA was applied in one eye in 10 nmol 0.9% NaCl drops (40 µl volume instilled) once a day for four consecutive days. The contralateral eye received the same volume of saline solution (0.9% NaCl). This method of delivery is particularly useful considering that the cornea is the most superficial ocular layer. Slit-lamp biomicroscopy was performed during the instillation process to evaluate possible changes in the cornea.

Corneal wounds were performed 10 h before the fourth siRNA instillation. After topical anesthesia (0.4% oxybuprocaine and 1% tetracaine; Alcon Cusi, Barcelona, Spain), corneal wounds were made to the epithelium of both eyes by applying a 3 mm disc of Whatman X1 paper soaked in n-heptanol (Sigma-Aldrich) as previously described [4]. Briefly, discs were place in the center of the cornea and left there for 30 s [18]. After removal of the disc, the eyes were washed with isotonic saline solution.

### Immunohistochemistry

Twelve, twenty-four, and thirty-six h after epithelium wounding, rabbits were euthanized with sodium pentothal and their eyes were enucleated. Corneas were dissected and fixed with 4% paraformaldehyde in 0.15 M PBS at 4 °C for 6 h. After fixation, corneas were embedded in Jung Tissue Freezing Medium (Leica Microsystems, Barcelona, Spain), and 10 μm sections were cut. The P2Y_2_-R immunocytochemical assay was performed as previously described for cells[6]. Briefly, sections were permeabilized with blocking solution (1X PBS, 3% BSA, Triton X-100, and 5% FBS) for 1 h to block non-specific binding. After washing with 1X PBS and 3% BSA, sections are incubated with primary goat polyclonal anti-P2Y_2_-R (1:50) or 1X PBS and 3% BSA for negative controls overnight at 4 °C. Sections were washed twice in 1X PBS and 3% BSA and incubated with the secondary antibody, donkey anti-goat IgG-FITC (1:200), for 1 h at room temperature. Finally, after washing in 1X PBS, slices were mounted with Vectashield mounting medium and observed under confocal microscope (Axiovert 200M; Carl Zeiss Meditec GmbH) equipped with a Pascal confocal module (LSM 5; Carl Zeiss Meditec GmbH). All images were managed with the accompanying Pascal software.

### Quantitative real-time RT–PCR

To validate the efficiency of *P2Y_2_-R* siRNA and its on-target effect, we measured the reduction of target mRNA level in treated corneas by quantitative RT-PCR (qRT–PCR). Corneal impression cytology samples were collected 48, 72, and 96 h after first siRNA instillation. After topical anesthesia (0.4% oxybuprocaine and 1% tetracaine; Alcon Cusi), a disc of cellulose acetate filter paper (HAWP304F0; Millipore, Bedford, EUA) was applied over the central corneal surface for 15 s. The cellulose acetate filter was immediately transferred into a tube containing 350 µl of RNA extraction reagent (RNeasy Mini Kit; Qiagen). All samples were stored at −80 °C until time of analysis. After vortex lysis/homogenization, we followed the manufacturer’s protocol. To avoid genomic DNA contamination, we performed the on-column DNA digestion step. For first-strand cDNA synthesis, 30 µl of total RNA was retrotranscribed using High Capacity cDNA RT kit (Applied Biosystems). Relative quantification of gene expression was performed using real-time RT–PCR on an ABI Prism 7300 Real-Time PCR System (Applied Biosystems) with Quantitect SYBR Green Kit (Qiagen) according to the manufacturer’s protocol. The following primers were used in real-time PCR amplification: *P2Y_2_-R* specific forward, 5′-TGG AGC CGT CTC TAA CCC TGA-3′ and *P2Y_2_-R* specific reverse, 5′-GCT GGC ACG CTG AAC CAG TA-3′). The level of *P2Y_2_-R* mRNA was normalized to the housekeeping gene, *HPRT1* (*HPRT1*-specific forward, 5′-CTG GCA AAA CAA TGC AGA CCT-3′ and *HPRT1*-specific reverse, 5′-GTC CTT TTC ACC AGC AGG CTT-3′). The remaining percentage of *P2Y_2_-R* mRNA was calculated using the Pfaffl method [19].

### In vivo migration assays

Rabbit corneas were treated with *P2Y_2_-R* siRNA #2 once a day for three days before the wounds were made, and the fourth dose of siRNA was applied 10 h after the wounds were made. Ap_4_A (100 μM) was topically applied to the wounds in Ap_4_A and siRNA+Ap_4_A corneas every 6 h (starting 10 h after the wound was made), and some animals were used as controls and was just treated with 0.9% saline.

To evaluate the closure of the wounds, these were stained with 2% fluorescein every 2 h with a gap of 10 h between the making of the original wounds until the beginning of measurements. This corresponds to the lag phase of the in vivo corneal wound healing process. The eyes were examined with a Topcon SL-8Z slit lamp (Topcon, Barcelona, Spain). Images were taken, managed, and analyzed with the IMAGEnet 2000 system (Topcon).

### Analysis of data

*P2Y_2_-R* down-regulation after siRNA transfection was determined by densitometric quantification of the P2Y_2_-R fluorescence signal in several images in four independent experiments. In each case, the results were expressed as mean fluorescence intensity (MFI)±SEM and represented in arbitrary units. A correlation between fluorescence intensities and the amount of receptors was performed.

To model the nonlinear decrease in wound area during epithelial healing, the constant velocity method previously described [20] was used with some modifications. Briefly, estimated migration rates (EMR) were determined by linear regression of the decrease in wound area during 10 h of measurements and were obtained by the slope of the regression line expressed as the percentage of area decrease per hour. The total time of wound closure (estimated healing time, EHT) was calculated by the extrapolation of the best fit of the regression line during the healing phase until there was 100% closure of each wound tested.

When we represent linear regression of the decrease in the wound area, data are expressed as a percentage of the initial wound width (at 0 h) to normalize variability in wounding from well to well and experiment to experiment following the same strategy as described by other authors [21,22].

EMR and EHT in treated and control wounds were compared using ANOVA test. Average values were expressed as mean±SEM. The levels of significance for the differences are indicated in each case in the figure captions.

## Results

### Cloning of rabbit *P2Y_2_-R*

To determine the role of P2Y_2_-R in Ap_4_A-induced rabbit corneal epithelial cell migration by siRNA technology, we previously cloned the rabbit *P2Y_2_-R* cDNA. Based on the conserved regions of coding sequences for human, rat, mouse, and pig *P2Y_2_-R*, degenerate primers were designed and used for initial RT–PCR experiments with total RNA isolated from SIRC cells. After sequencing PCR products, a new pair of primers was synthesized and used to obtain contiguous sequence information. Finally, the sequences obtained from RT–PCRs were used to design specific primers to rabbit *P2Y_2_-R* to facilitate amplification of 5′-end cDNA (5′-RACE). Assembly of the sequences obtained yielded a sequence of 1,094 bp (GenBank EU886321). Protein database searches revealed that the deduced sequence had the highest amino acid identity with human P2Y_2_-R (93% identity; see Figure 1B).

### Efficiency of siRNA transfection into SIRC cells

Preliminary studies were performed to confirm siRNA transfection. Cells were incubated for 24 h with 30 nM Cy3-labeled non-silencing siRNA. Cultures were then washed, fixed, and visualized by fluorescence microscopy. The presence of Cy3-positive cells indicated successful siRNA transfection (data not shown). In addition, the cell viability assay demonstrated that under these transfection conditions, SIRC cells were not significantly affected (81% cell viability).

### Safety of in vivo siRNA delivery

To confirm the absence of corneal changes after frequent instillations of siRNA, we performed a biomicroscopic examination of the treated eyes. Neither corneal inflammation, cataract, or any other ocular alteration were observed throughout siRNA administration (results not shown).

### Inhibition of *P2Y_2_-R* expression by siRNA

To test the efficacy of synthesized siRNAs targeting *P2Y_2_-R*, immunocytochemistry (ICC), immunohistochemistry (IHC), and qRT–PCR assays were performed.

**Figure 2:**
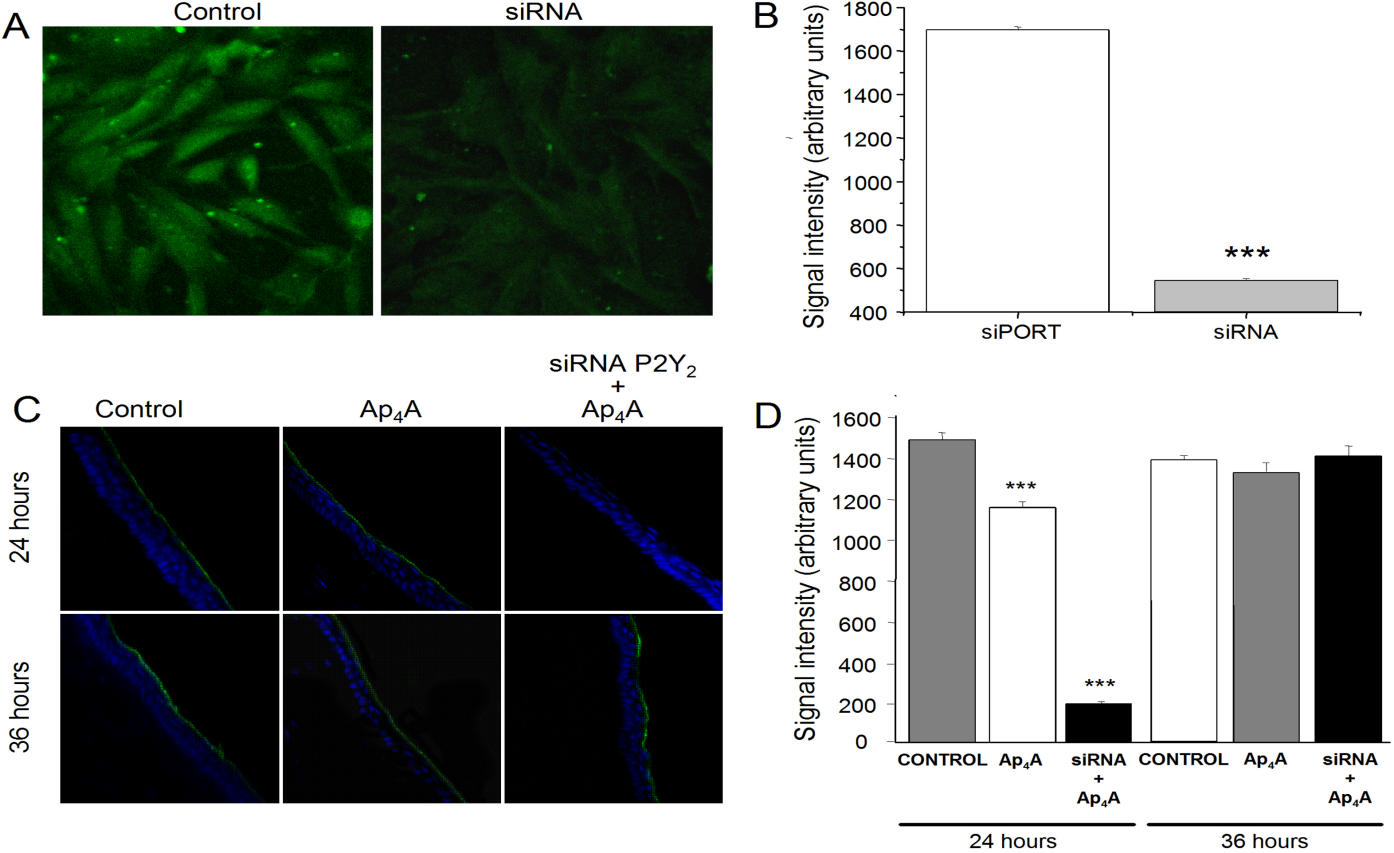
P2Y_2_-R immunostaining of transfected cells and treated corneas. **A**: SIRC cells were incubated for 72 h with transfection reagent alone (control) or with *P2Y_2_-R* siRNA #2 and then processed for ICC (40X magnification). **B**: The chart shows P2Y_2_-R staining intensity quantification of control and *P2Y_2_-R* siRNA transfected cells 72 h post-transfection. **C**: A series of micrographs shows the P2Y_2_-R signal in corneas treated with 0.9% saline,  100 μM Ap_4_A, and siRNA+100 μM Ap_4_A while we can observed the nuclear staining for DAPI in blue (40X). **D**: The graph shows the P2Y_2_-R intensity signal in the three different treatments at 24 h and 36 h after wounding. Three asterisks mean p<0.001 when compared to the control. Green fluorescence (FITC) localizes P2Y_2_-R.

Cells were transfected with 300 nM *P2Y_2_-R* siRNA #1, 300 nM *P2Y_2_-R* siRNA #2, or the transfection reagent alone (control) for 72 h and then processed for ICC. Staining for P2Y_2_ receptor was reduced in cells incubated with both *P2Y_2_-R* siRNA #1 (data not shown) and *P2Y_2_-R* siRNA #2 transfected cells. The P2Y_2_-R reduction obtained with *P2Y_2_-R* siRNA #2 (Figure 2A) was greater than the observed reduction for *P2Y_2_-R* siRNA #1 (result not shown). Therefore, according to the in vitro effect of siRNA on migration rate, we chose *P2Y_2_-R* siRNA #2 for the in vivo studies.

A qualitative measure of the remaining P2Y_2_-R was performed from the fluorescence signal intensity in both the control and siRNA-transfected cells as described in Methods. Figure 2B shows a plot of the amount of P2Y_2_-R receptor in each case. The amount of P2Y_2_-R in the control cells was 1698.82±12.66 while in *P2Y_2_-R* siRNA #2-transfected cells, the amount of P2Y_2_-R was 547.04±6.78 (p<0.0001). These data indicate that the P2Y_2_-R levels were significantly reduced to approximately 32% of control levels by *P2Y_2_-R* siRNA #2.

The effect of the siRNA against P2Y_2_-R was tested in living animals. Prior to that assay, corneas were treated to identify the localization of P2Y_2_-R. As we can observe in Figure 2C, P2Y_2_-R signaling is localized only in the outer layer of the epithelium, this signal being higher in control corneas than in Ap_4_A-treated corneas 24 h after wounding (1481.93±34.20 for control and 1149.46±28.04 for Ap_4_A, p<0.001; Figure 2C, upper panel). Thirty-six h after the wounds were performed, P2Y_2_-R staining was similar to the 24 h results (1400.05±19.49 for control and 1347.70±19.95 for Ap_4_A; Figure 2C, lower panel). When the experiment was performed on living animals, the profile of P2Y_2_-R expression in siRNA #2-treated animals presented a marked reduction 24 h after the wound was performed (189.36±10.73, p<0.001) compared to the control and Ap_4_A-treated corneas. The analysis for a longer period of time (36 h after the wound) demonstrated a full recovery of the P2Y_2_ signaling, similar to the control (1424.14±14.1; Figure 2D).

Concerning the results 12 h after the wound, P2Y_2_-R staining in the three different treatments were similar to those obtained for 24 h after wounding (data not shown). These results indicate that silencing P2Y_2_-R in our model was detected 24 h after the wound was performed. Nevertheless, P2Y_2_-R signal was again visible 36 h after the wound had been made.

**Figure 3:**
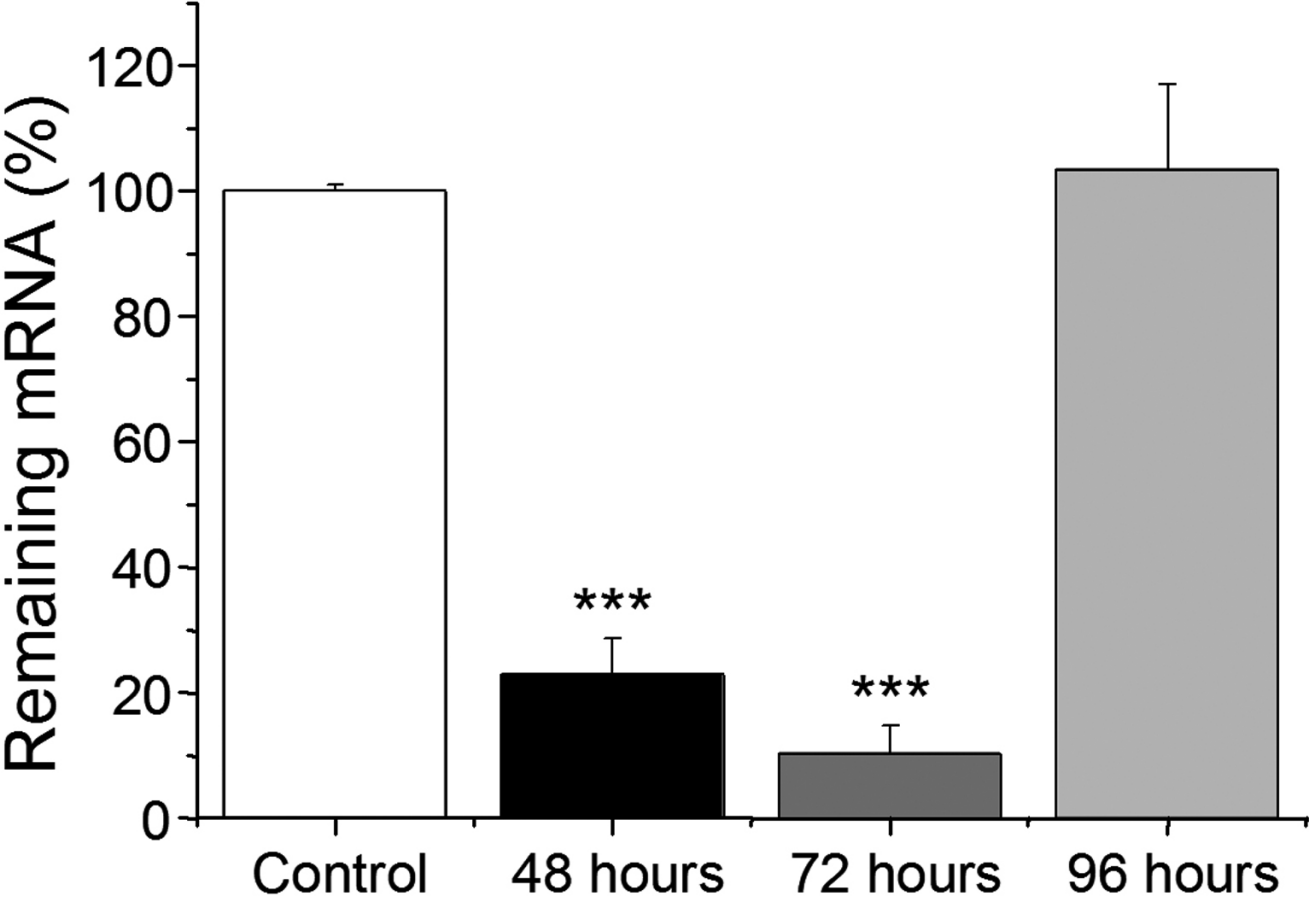
Quantification of *P2Y_2_-R* mRNA levels by qRT–PCR analysis. Corneal impression cytology samples were collected at 48, 72, and 96 h after the first siRNA instillation and then submitted to total RNA isolation. After retrotranscription, we measured *P2Y_2_-R* mRNA knockdown in corneas treated with siRNA #2. Values derived from qRT–PCR results from untreated corneas are set as 100%, and the relative expressions of corneas treated with siRNA are indicated. Three asterisks indicate p<0.001 when compared to the control.

Quantification of siRNA #2 silencing efficiency was also performed by qRT–PCR. As shown in Figure 3, significant *P2Y_2_-R* mRNA knockdown was observed after 48 and 72 h of the first instillation (23.00%±5.65% and 10.36%±4.47% of *P2Y_2_-R* mRNA remaining, respectively; p<0.001). However, at 96 h (36 h after wound injury), the effect at the mRNA level disappears.

### *P2Y_2_-R* siRNA counteract the effect of Ap_4_A on corneal wound healing

In a previous work, we have demonstrated the Ap_4_A-accelerating effect on rabbit corneal wound healing and provided pharmacological evidence for P2Y_2_-R mediation [6]. To know the role of rabbit P2Y_2_-R in Ap_4_A-re-epithelialization process, a migration assay was performed on cells transfected with *P2Y_2_-R* siRNA #1 and *P2Y_2_-R* siRNA #2.

**Figure 4:**
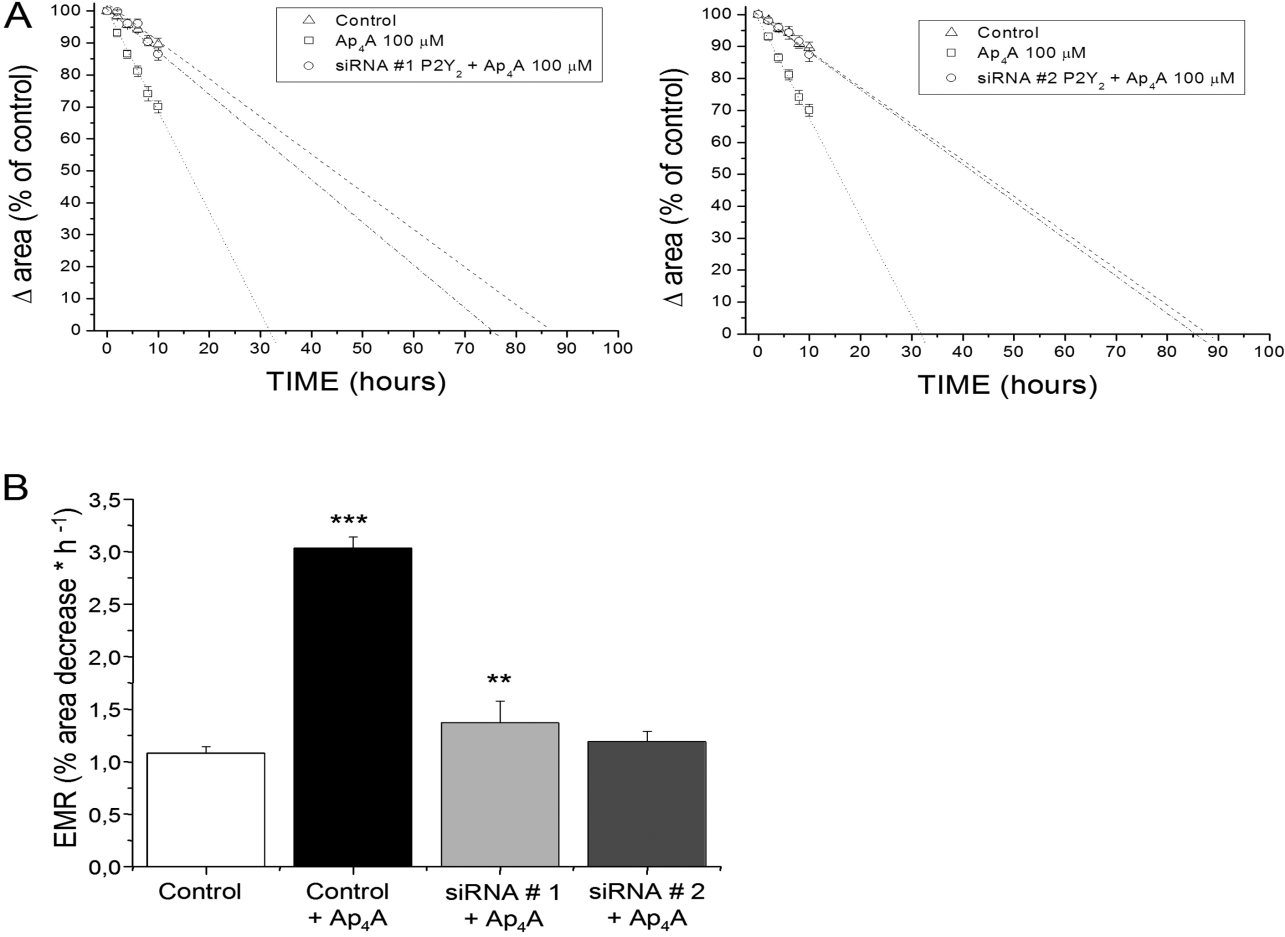
Effect of *P2Y_2_-R* siRNA on Ap_4_A-induced migration of corneal epithelial cells. SIRC cells were incubated for 72 h with transfection reagent alone (control), *P2Y_2_-R* siRNA #1, or *P2Y_2_-R* siRNA #2 and then wounded with a pipette tip in the presence or absence of Ap_4_A. The graphs (**A** and **B**) show the variation of the wounded area versus time and the estimated migration rates (EMR) of cells transfected with siRNA 1 and siRNA 2, respectively, in the presence of Ap_4_A (*P2Y_2_-R* siRNA+Ap_4_A). Ap_4_A accelerated the rate of healing in cells transfected with transfection reagent alone (control+Ap_4_A) compared with the rate of both *P2Y_2_-R* siRNA-transfected cells in the presence of Ap_4_A (*P2Y_2_-R* siRNA+Ap_4_A). Three asterisks indicate p<0.0001 when compared to the control, and two asterisks indicate p<0.001 when compared to Ap_4_A alone.

Seventy-two h post-transfection, cells were wounded with a pipette tip in the presence or absence of Ap_4_A. Graphics in Figure 4A show the variation of the wounded area versus time, and Figure 4B shows the estimated migration rates (EMR) of control cells (treated with the transfection reagent, siPORT, alone), cells treated with Ap_4_A (100 µM) and *P2Y_2_-R_ 2_* siRNA #1-, and *P2Y_2_-R* siRNA #2-transfected cells treated with Ap_4_A (100 µM; repeated tour times for each siRNA). Ap_4_A significantly increased the estimated migration rate (3.03%±0.11%) when compared to the control (1.08%±0.06%; p<0.0001). This represents a concomitant decrease in the estimated healing time (EHT) with closure of the wound occurring 56 h earlier than in the absence of any added substance. In the case of *P2Y_2_-R* siRNA #1+Ap_4_A-treated cells, we found an EMR decrease (1.37%±0.21%) and the concomitant EHT increase (74 h) when compared with just Ap_4_A-treated cells. On the other hand, treatment with *P2Y_2_-R* siRNA #2 and Ap_4_A caused a high decrease in EMR (1.19%±0.10%; p<0.001) when compared to just Ap_4_A-treated cells and a concomitant increase in EHT (86 h), which were similar to the EMR and EHT values found in control wounds (Figure 4B). All these results confirm the *P2Y_2_-R* silencing efficiency of *P2Y_2_-R* siRNA #2 and also the involvement of P2Y_2_-R in the Ap_4_A re-epithelialization process.

**Figure 5:**
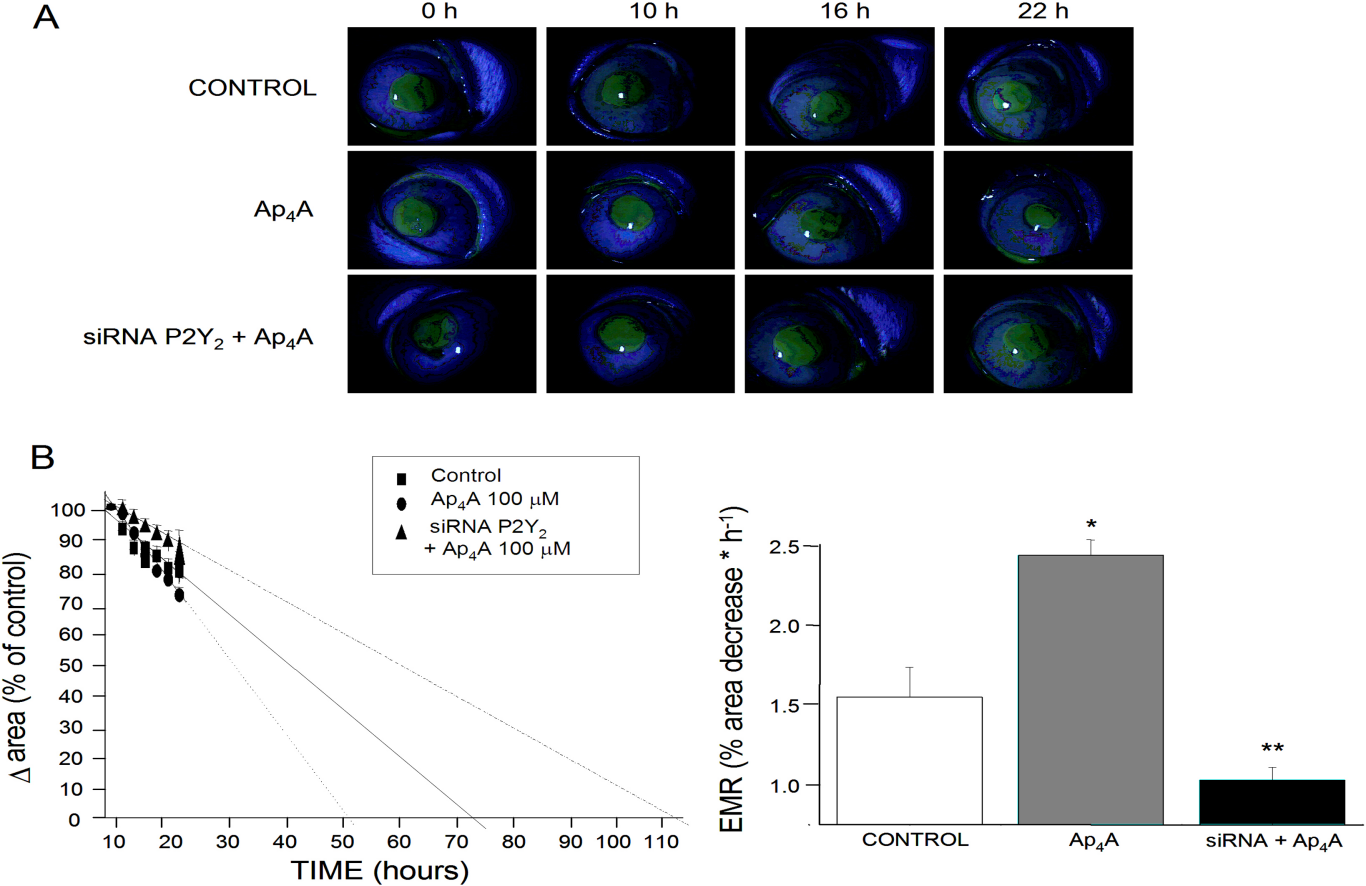
Effect of *P2Y_2_-R* siRNA on Ap_4_A-mediated corneal wound healing. **A**: A representative sequence of the progress in the corneal wound in the three different treatments (0.9% saline, 100 μM Ap_4_A, and siRNA+100 μM Ap_4_A) is shown. **B**: The graphs show the variation of the wounded area versus time and the estimated migration rates (EMR) in the three treatments. An asterisk means p<0.05 when compared to the control, and a double asterisk indicates p<0.01 when compared to the control.

After verifying the effect of *P2Y_2_-R* siRNA #2 transfection on SIRC cells, we wanted to correlate this effect with siRNA in vivo migration assays. Figure 5A shows a series of images where we can demonstrate the closure of corneal wounds after the three different treatments (control with 0.9% saline, 100 μM Ap_4_A, and siRNA+100 μM Ap_4_A). We can observe that after the challenge with Ap_4_A, the migration rate is accelerated compared to the control wounds while the treatment with siRNA+Ap_4_A produces a dramatic delay in migration.

This can also be observed in Figure 5B where a plot for variation in wounded area versus time and a plot of EMR for the three treatments are shown. Ap_4_A (100 μM) significantly increased EMR (2.44%±0.10%; p<0.05) in contrast to the control wounds (1.55%±0.19%) with the concomitant decrease in EHT (from 76 h for control to 55 h for Ap_4_A). On the other hand, the pre-treatment with siRNA against *P2Y_2_-R* produced a long delay in EMR (1.03%±0.08%; p<0.05), increasing the time for closure to 109 h. All these data reveal the involvement of P2Y_2_-R on the in vivo migration of epithelial cells triggered by Ap_4_A.

## Discussion

Normal visual function depends on the ability of the cornea to form a physical barrier against infections and mainly to form a smooth and transparent refractive surface. These corneal properties are sustained by the renewal/wound healing process. The involvement of P2Y_2_-R and P2Y_4_-R in corneal wound healing has been previously suggested by our and other groups through pharmacological tools [4,6,23,24]. Furthermore, the presence of these receptors on corneal epithelial cells has been reported by immunohistochemical and in situ hybridization techniques [25,26].

The pharmacological profile of P2Y_2_-R subtypes can vary, depending on the species studied. Thus, there is a lack of selective agonists/antagonists to prove the participation of a specific P2Y-R in a cellular process [7-9].

In the current study, we extended our inquiry to determine the role of P2Y_2_-R in Ap_4_A-re-epithelialization process through RNAi technology. The RNAi tool has been successfully used for years to study gene function in the in vitro and in vivo models. In this way, siRNAs against P2 receptors have been previously used to understand its physiologic roles [27-33].

Two siRNAs were designed from the previously cloned rabbit *P2Y_2_-R* cDNA. Efficiency of siRNA transfection and gene silencing was confirmed in SIRC cells. *P2Y_2_-R* siRNA #2- protein knockdown was clearly observed by ICC assays. In addition, both *P2Y_2_-R* siRNA #1 and *P2Y_2_-R* siRNA #2 reduce migration rates only in cells wounded and treated with Ap_4_A. These results confirm the specific effect (on target) of our *P2Y_2_-R* siRNAs.

We chose *P2Y_2_-R* siRNA #2 for in vivo assays because of its greatest reduction in migration rate of cells treated with Ap_4_A.

In the eye, direct application of siRNAs has been performed and its potential use as an ocular therapeutic agent has also been demonstrated [10,12]. To achieve high siRNA silencing efficacy in the in vivo models, it is critical not only to design specific siRNAs but also to choose the most appropriate route of delivery. Between the ocular routes of administration, we chose topical instillation because of our target tissue (cornea). Topical instillation is a noninvasive technique and lacks toxicity. Due to the corneal-protective characteristics, we performed frequent instillations. However, we did not observe changes in the cornea by slit-lamp biomicroscopy.

Efficiency of P2Y_2_-R silencing was also confirmed in living animals by IHC and quantitative RT-PCR (qRT-PCR) analysis. In addition, protein and mRNA reduction, ruled out a possible microRNA (miRNA)-related translational mechanism [10,34,35].

Our IHC results reveal that after wounding, P2Y_2_-R staining in Ap_4_A-treated wounds is less intense than in control wounds. As with many other agonists (for example, insulin), when Ap_4_A binds to its receptor, P2Y_2_-R, on the surface of a cell, the Ap_4_A-P2Y_2_-R complex undergoes endocytosis and is subsequently attacked by intracellular lysosomal enzymes. The internalization of the Ap_4_A molecules provides a pathway for degradation of the Ap_4_A-P2Y_2_-R complex as well as for regulation of the number of sites that are available for binding on the cell’s surface. The rate of synthesis of new receptors within the endoplasmic reticulum and their insertion in the plasma membrane does not keep pace with their rate of destruction. Over time, this self-induced loss of target cell receptors for Ap_4_A reduces the target cell’s sensitivity to the elevated dinucleotide concentration [36,37].

In vivo corneal wound healing is a process that occurs in three main steps: lag phase (from 0 h to 10 h after the wound), migration phase (until 24–36 h after the wound), and mitosis phase (lasting from 36 h after the wound to weeks) [38]. In this manuscript, we have investigated the role of P2Y_2_-R in the Ap_4_A acceleration of the migration rate after corneal wound healing both in vitro and in vivo. The tested siRNA performed its effect in the first 36 h after the wound, a period that correlates with the migration phase that we have investigated in this manuscript. Altogether, these results confirm the role of P2Y_2_-R in corneal wound healing. As indicated in our results, a selective siRNA against *P2Y_2_-R* significantly blocked the ability of the dinucleotide Ap_4_A to accelerate the rate of re-epithelialization after corneal scratch.

Preliminary experiments performed in our laboratory established that the application of Ap_4_A induces an increase in the rate of migration [4,6]. In our earlier study and according to our pharmacological profile, we suggested a P2Y_2_-R mechanism. In the present work, it seems clear that wound healing effect evoked by Ap_4_A is due to activation of P2Y_2_-R.

Demonstrating the presence and the involvement of P2Y_2_-Rs in the corneal wound healing process will allow us to develop compounds for the treatment of corneal wounds and even for corneal repair after refractive surgery.

It is clear that selective and stable compounds are necessary to produce a positive and sustained wound healing process. This is even more relevant if we take into consideration the presence of enzymes cleaving nucleotides in the ocular surface and the presence of other P2 receptors delaying corneal wound healing [39]. In this context, we suggested in a previous work possible P2Y_2_-R/P2Y_6_-R switches, which control the transition from wound healing phase 2 to phase 3 [4,6]. More experiments will be necessary to confirm this hypothesis and a possible approach is the use of multi/individual-target siRNA.

In summary, the cloning and the design of a *P2Y_2_-R* siRNA has allowed us to demonstrate the involvement of this receptor in a relevant process such as corneal wound healing. This demonstrates that the use of siRNA to silence proteins is an interesting tool for receptors that do not have selective antagonists to prove their participation in biochemical and physiologic processes.
